# A deafening silence: a lack of data and reproducibility in published bioacoustics research?

**DOI:** 10.3897/BDJ.7.e36783

**Published:** 2019-10-30

**Authors:** Ed Baker, Sarah Vincent

**Affiliations:** 1 Natural History Museum, London, United Kingdom Natural History Museum London United Kingdom; 2 University of York, York, United Kingdom University of York York United Kingdom

**Keywords:** bioacoustics, open data, reproducibility, sound libraries, acoustic vouchers

## Abstract

A study of 100 papers from five journals that make use of bioacoustic recordings shows that only a minority (21%) deposit any of the recordings in a repository, supplementary materials section or a personal website. This lack of deposition hinders re-use of the raw data by other researchers, prevents the reproduction of a project's analyses and confirmation of its findings and impedes progress within the broader bioacoustics community. We make some recommendations for researchers interested in depositing their data.

## Introduction

The importance of sharing the datasets used in biological research has been discussed recently by a number of authors, for example, in ecology (e.g. Poisot et al. 2013, Kenall et al. 2014), phylogenetics (e.g. Magee et al. 2014, Stoltzfus et al. 2012) and behaviour (e.g. Caetano and Aisenberg 2014). These authors list several benefits of sharing data, including the opportunity to create future collaborations and clarification of authorship. There can also be ethical considerations, including the use of public funds to generate these datasets. Another significant reason for sharing datasets underpinning research is to ensure that those findings are reproducible, a fact which advocates for open science have discussed well before the recent 'reproducibility crisis' in psychology (Open Science Collaboration 2015).

The datasets used in bioacoustic research vary in scale from a single short recording to continuous recordings at a site over several years. These recordings may be used to identify (e.g. Heller and Baker 2017) or describe (e.g. Dring 1983) species new to science and to facilitate long-duration biological surveys (e.g. Eichinski and Roe 2017). Additionally, recordings may be used to design automated surveys that do not themselves make recordings (e.g. Bennett et al. 2015).

## Material and methods

The twenty most recently published articles (as of the end of 2017) covering bioacoustic topics were selected from each of the journals Bioacoustics, ZooKeys, ZooTaxa, Journal of Animal Behaviour and Marine Mammal Science. Primary research articles were identified using the search terms "acoustics" and "bioacoustics" on the journal's website; only articles making use of recorded sounds were selected. The journals chosen cover a subject-specific journal (Bioacoustics), a modern semantically enhanced (Penev et al. 2010) journal (ZooKeys), a taxonomic mega-journal (ZooTaxa), a journal with a broader zoological scope (Journal of Animal Behaviour) and a taxonomically focussed journal (Marine Mammal Science).

Both authors independently examined the papers for evidence that the underlying sound recordings were available in either physical or digital repositories. Two scoring systems were used to categorise the papers:

- The first identifies those papers that define a repository for sound recordings;
- The second differentiates between the type of deposition (repository, personal website, supplementary material) and whether the recordings deposited are the complete set or a representative sample.

## Data resources

A CSV file of the papers analysed (including their DOIs) and scored values is available in Suppl. materials 1, 2, full Crossref metadata for the articles is available in Suppl. materials 3, 4 .

## Results

**Figure 1.**
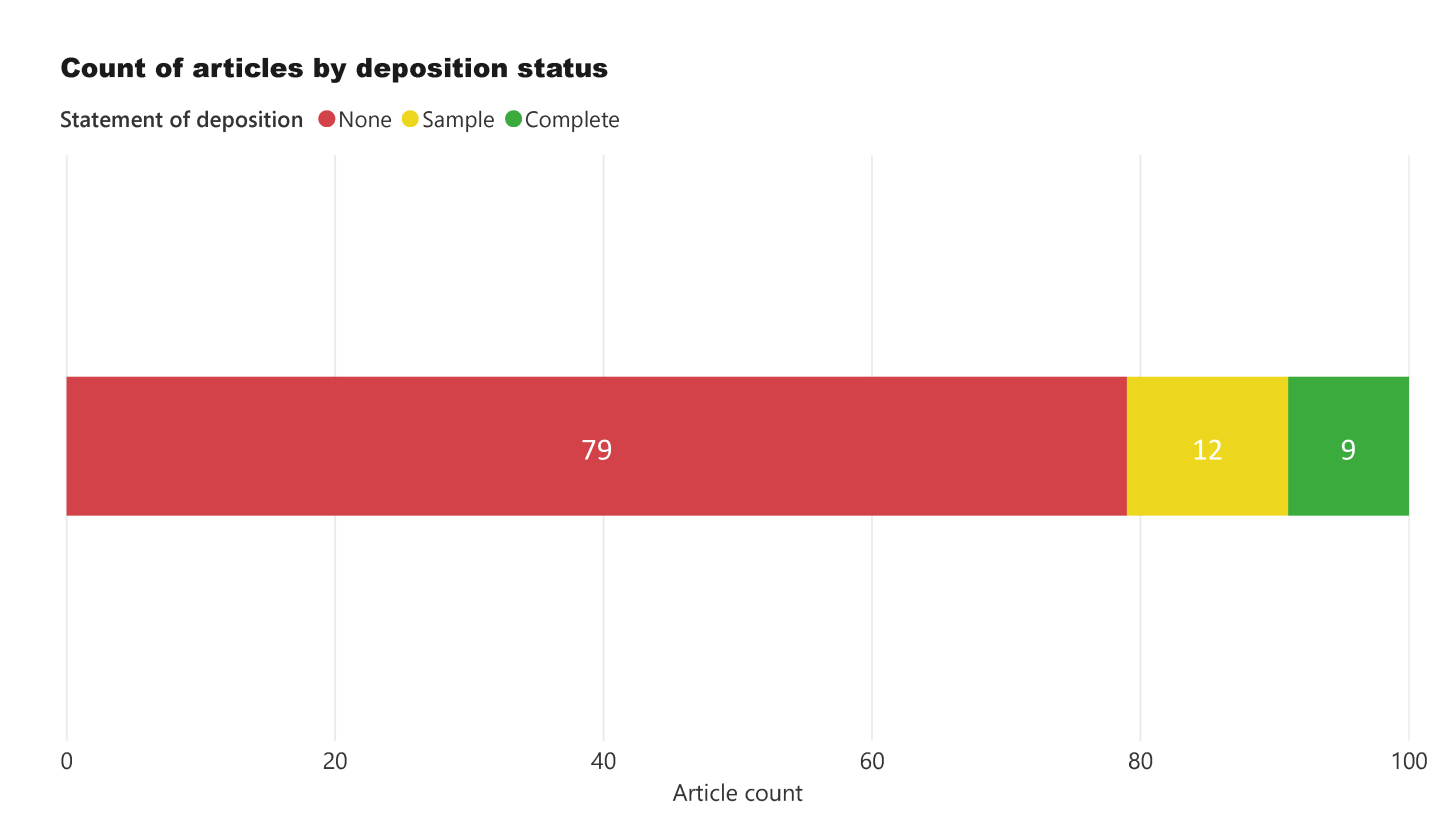
Deposition status of all articles in the study.

**Figure 2.**
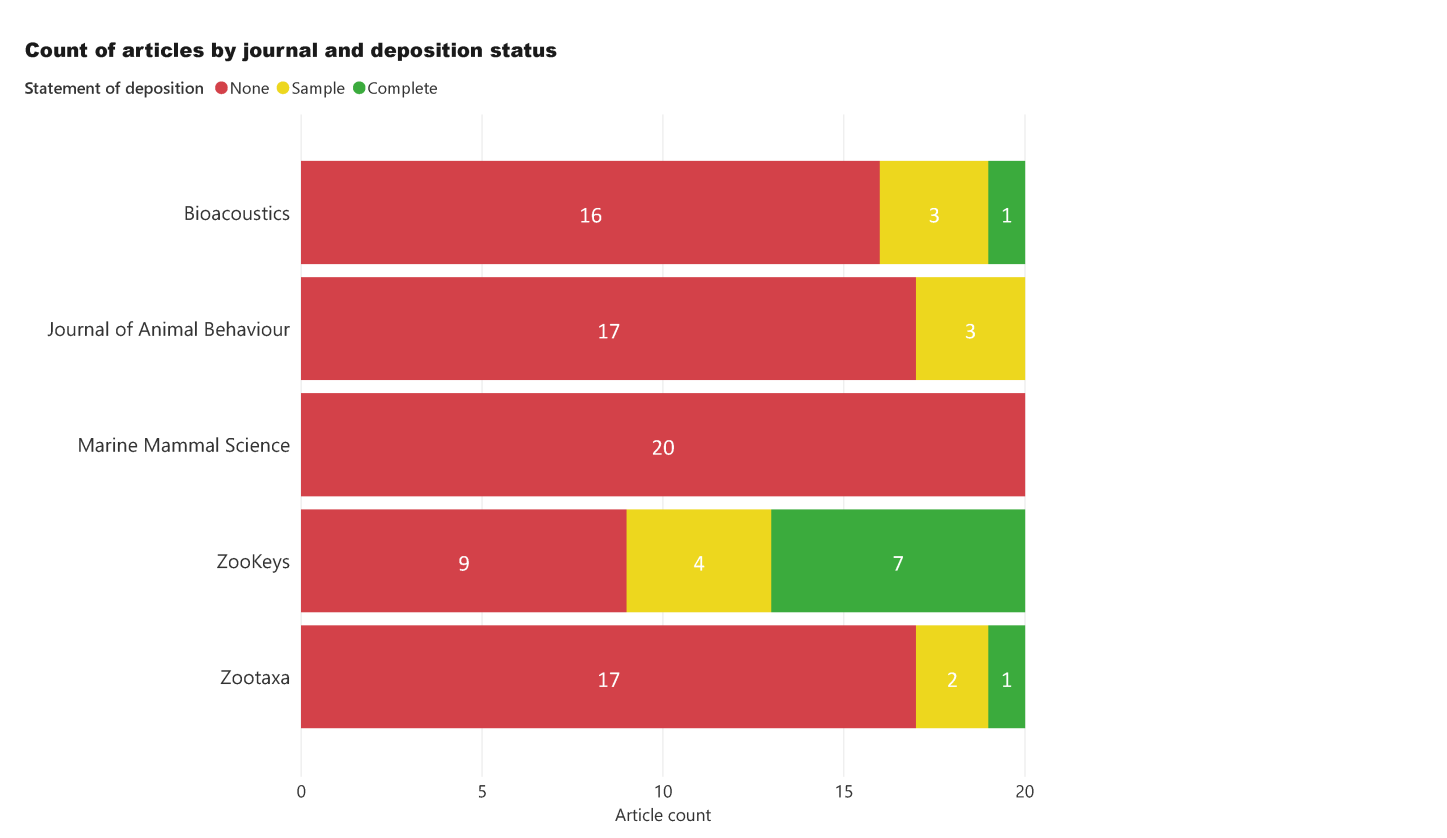
Breakdown of article deposition status by journal.

Out of all the articles in the study, 79% did not deposit any sound recordings, 12% deposited a sample of the studied data and only 9% deposited the full acoustic dataset (Fig. 1). Of those articles depositing the full dataset, 78% were from the same journal (ZooKeys). ZooKeys was the only journal to achieve a deposition rate of over 50% when considering both complete and sample depositions (Fig. 2).

**Figure 3.**
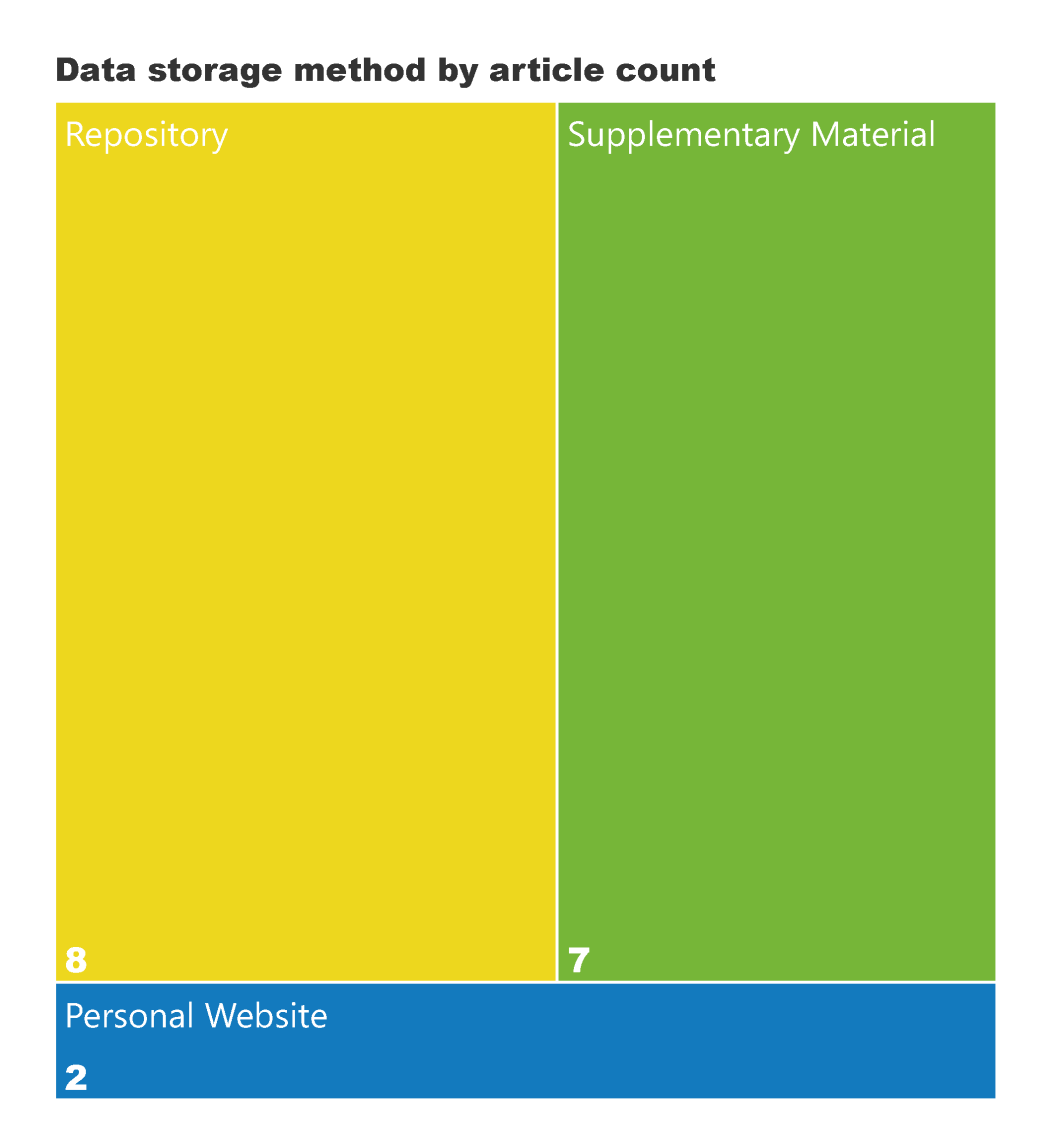
Deposition method for papers that deposit data.

Where complete data were deposited, the majority used a repository (8%) or the supplementary materials of the journal article (7%). Only 2% used a personal website (Fig. 3).

## Discussion

It can be seen from these results that only a minority (21%) of the published studies analysed deposit the sound recordings on which their results are based. In addition, even when authors claim deposition, there can still be problems. Issues identified in this study include not depositing recordings, broken URLs and providing the wrong URL for a repository (for a list see Suppl. material 1).

This lack of deposition is potentially problematic for the reproducibility of research and also hinders the re-use of recordings by other researchers. Given widespread lack of deposition described in this paper, below we provide some recommendations which researchers may use to improve the accessibility of their bioacoustic data.

### Reproducibility

It may be argued for well-studied, easily identified taxa with relatively stable taxonomy, that there is no need to deposit recordings and that a well-documented methodology is sufficient to ensure reproducibility. The reasons why this does not extend to all taxa have been discussed previously in the context of voucher specimens for biodiversity and community ecology by Turney et al. (2015) and for phylogenetics by Pleijel et al. (2008). As many bioacoustic signals are unique to species, they can, in many cases, be considered to be surrogates for voucher specimens. As a minimum, we therefore recommend deposition of a sample of voucher sound recordings. Where it is feasible to collect voucher specimens and sound recordings, linking recordings to specimens in museum collections provides an even more robust identity for the organisms studied and allows the published study to remain relevant, even if the species studied is later found to be two or more species.

### Making Data Available

Dedicated bioacoustic repositories often have the advantage of integrating with other components of the biodiversity informatics landscape, for example using Darwin Core (Wieczorek et al. 2012) to provide species locality data to the Global Biodiversity Informatics Facility (GBIF). This integration with external aggregators adds additional impact to the datasets that are shared; however it is not universally adopted by bioacoustic repositories. GBIF is perhaps the most prominent tool for searching for recordings of species across the datasets of multiple institutions and we strongly encourage institutions holding bioacoustic data to contribute.

Riede (2018) discusses potential depositories for the singing Orthoptera, the Macaulay Library and xeno-canto have large collections of birdsong. The BioAcoustica database (Baker et al. 2015b) accepts recordings of any species in addition to soundscape recordings and makes data available to the Encyclopedia of Life and GBIF (Baker et al. 2015a) . The choice of repository is down to individual researchers and the taxa they work with; however, considerations may include whether these repositories make the original sound files available online (instead of just MP3s which may not contain all relevant acoustic data), whether the files can be openly licensed (e.g. using Creative Commons) and whether off-site backups are provided to protect against data loss (e.g. Dena et al. 2018). Some repositories (e.g. the Data Portal at the Natural History Museum, London; Scott et al. 2019) provide DataCite DOIs for contributed datasets, allowing for individual datasets to be cited.

Many of the advances in large-scale ecoacoustics will rely on large datasets that are labelled suitably for machine learning algorithms. While these datasets are becoming available for well-studied groups of organisms (Morfi et al. 2019), expanding this to less well-studied taxa will require widespread data sharing to obtain comparable datasets in a reasonable timeframe.

## Conclusions

More work needs to be done by the bioacoustics community to create an environment where the data underpinning research are made available, ideally using the FAIR Data Principles of being findable, accessible, interoperable and reusable. A discussion of the FAIR principles for scientific data is provided in Wilkinson et al. (2016). The loss of data due to natural or anthropogenic causes (e.g. Dena et al. 2018) is reduced by having an independent backup in an external location.

### Recommendations

The recommendations below are based on issues we have identified in the research for this paper.

1. The use of a repository (either a formal repository or institutional data portal) is recommended for bioacoustic recordings to aid with the findability and accessibility components of the FAIR data principles. Consideration should be given to the long-term sustainability of the repository (e.g. institutional support), how that repository connects to the wider biodiversity informatics landscape and the formats in which it makes audio files available (some repositories only make lossily-compressed MP3 files available to end users even though they have WAV files available internally).
2. The repository where recordings will be deposited should be identified before a paper is submitted. Working with the repository will allow for URLs to individual recordings (or sets of recordings) to be included within the paper, instead of a generic reference to the repository that the end-user must then search or browse to uncover the recordings. Communication with the repository prior to publication should eliminate any issues of providing incorrect URLs in published works. Depositing recordings prior to submission will also prevent instances of papers claiming submission to a repository, but the authors then forget to make such depositions.
3. Repositories may allow for an embargo on the public release of recordings until a paper is published and/or for a time-limited period subsequently.
4. The use of the Supplementary Materials section of journals is not recommended for audio deposition, as access may be limited to journal subscribers and they are not at present discoverable via aggregators such as GBIF.
5. Repositories should have a mechanism to prevent link-rot from changing URLs, ensuring that cited URLs are always resolvable.
6. Use of an open licence such as Creative Commons allows data to be re-used easily by other researchers.

## Supplementary Material

CF640A9C-07E2-52AE-ACB6-20C24E508DF9 10.3897/BDJ.7.e36783.suppl1 Supplementary material 1 Scoring of Articles (CSV) Data type: references
Brief description: The scoring of the articles used in this study.
File: oo_304447.csv https://binary.pensoft.net/file/304447 Ed Baker; Sarah Vincent

CA4CCC8E-FA79-59CF-81A6-A65967CB0632 10.3897/BDJ.7.e36783.suppl2 Supplementary material 2 Scoring of Articles (Excel) Data type: references
Brief description: The scoring of articles used in this study.
File: oo_348433.xlsx https://binary.pensoft.net/file/348433 Ed Baker; Sarah Vincent

6B3734A5-C9A4-5B3B-BE92-D3CEDB0B8A78 10.3897/BDJ.7.e36783.suppl3 Supplementary material 3 Crossref Metadata for papers analysed (CSV) Data type: references
Brief description: Full metadata for the articles analysed.
File: oo_304446.csv https://binary.pensoft.net/file/304446 Sarah Vincent

66CAFFF3-6CDD-58B4-907D-AD30F4F9272F 10.3897/BDJ.7.e36783.suppl4 Supplementary material 4 Crossref Metadata for papers analysed (Excel) Data type: references
Brief description: Full metadata for the articles analysed.
File: oo_348434.xlsx https://binary.pensoft.net/file/348434 Ed Baker; Sarah Vincent

## References

[B5238898] Baker E., Price B., Rycroft S., Villet M (2015). Global Cicada Sound Collection I: Recordings from South Africa and Malawi by B. W. Price & M. H. Villet and harvesting of BioAcoustica data by GBIF. Biodiversity Data Journal.

[B3756618] Baker E., Price B. W., Rycroft S. D., Hill J., Smith V. S. (2015). BioAcoustica: a free and open repository and analysis platform for bioacoustics. Database.

[B5238888] Bennett Wil, Chesmore David, Baker Edward (2015). Speckled bush cricket data logger - Project Report. Figshare.

[B4604165] Caetano Daniel S., Aisenberg Anita (2014). Forgotten treasures: the fate of data in animal behaviour studies. Animal Behaviour.

[B4903304] Dena Simone, Rebouças Raoni, Augusto-Alves Guilherme, Toledo Luís Felipe (2018). Lessons from recordings lost in Brazil fire: deposit and back up. Nature.

[B5238843] Dring Julian (1983). Some new frogs from Sarawak. Amphibia-Reptilia.

[B5238878] Eichinski Philip, Roe Paul (2017). Clustering and visualization of long-duration audio recordings for rapid exploration avian surveys. 2017 IEEE 13th International Conference on e-Science (e-Science).

[B4611182] Heller K. -G., Baker Ed (2017). From an old sound recording to a new species in the genus *Horatosphaga* (Orthoptera: Tettigonioidea: Phaneropterinae: Acrometopini). Zootaxa.

[B4604155] Kenall Amye, Harold Simon, Foote Christopher (2014). An open future for ecological and evolutionary data?. BMC Evolutionary Biology.

[B4604175] Magee Andrew F., May Michael R., Moore Brian R. (2014). The dawn of open access to phylogenetic data. PLoS ONE.

[B5382538] Morfi Veronica, Bas Yves, Pamuła Hanna, Glotin Hervé, Stowell Dan (2019). NIPS4Bplus: a richly annotated birdsong audio dataset. PeerJ Computer Science.

[B4604185] Collaboration Open Science (2015). Estimating the reproducibility of psychological science. Science.

[B5414337] Penev Lyubomir, Agosti Donat, Georgiev Teodor, Catapano Terry, Miller Jeremy, Blagoderov Vladimir, Roberts David, Smith Vincent, Brake Irina, Ryrcroft Simon, Scott Ben, Johnson Norman, Morris Robert, Sautter Guido, Chavan Vishwas, Robertson Tim, Remsen David, Stoev Pavel, Parr Cynthia, Knapp Sandra, Kress W. John, Thompson Frederic, Erwin Terry (2010). Semantic tagging of and semantic enhancements to systematics papers: ZooKeys working examples. ZooKeys.

[B5382500] Pleijel F., Jondelius U., Norlinder E., Nygren A., Oxelman B., Schander C., Sundberg P., Thollesson M. (2008). Phylogenies without roots? A plea for the use of vouchers in molecular phylogenetic studies. Molecular Phylogenetics and Evolution.

[B4604131] Poisot Timothee, Mounce Ross, Gravel Dominique (2013). Moving toward a sustainable ecological science: don't let data go to waste!. Ideas in Ecology and Evolution.

[B5238833] Riede Klaus (2018). Acoustic profiling of Orthoptera: present state and future needs. Journal of Orthoptera Research.

[B5238807] Scott Ben, Baker Ed, Woodburn Matt, Vincent Sarah, Hardy Helen, Smith Vincent S (2019). The Natural History Museum Data Portal. Database.

[B4604141] Stoltzfus Arlin, O'Meara Brian, Whitacre Jamie, Mounce Ross, Gillespie Emily L, Kumar Sudhir, Rosauer Dan F, Vos Rutger A (2012). Sharing and re-use of phylogenetic trees (and associated data) to facilitate synthesis. BMC Research Notes.

[B5382466] Turney Shaun, Cameron Elyssa R., Cloutier Christopher A., Buddle Christopher M. (2015). Non-repeatable science: assessing the frequency of voucher specimen deposition reveals that most arthropod research cannot be verified. PeerJ.

[B5238819] Wieczorek J, Bloom D, Guralnick R, Blum S, Doering M, Giovanni R, Robertson T, Vieglaiset D (2012). Darwin Core: An evolving community-developed biodiversity data standard. PLoS ONE.

[B5240672] Wilkinson Mark D, Dumontier Michel, Aalbersberg I Jsbrand Jan, Appleton Gabrielle, Axton Myles, Baak Arie, Blomberg Niklas, Boiten Jan-Willem, da Silva Santos Luiz Bonino, Bourne Philip E, Bouwman Jildau, Brookes Anthony J, Clark Tim, Crosas Mercè, Dillo Ingrid, Dumon Olivier, Edmunds Scott, Evelo Chris T, Finkers Richard, Gonzalez-Beltran Alejandra, Gray Alasdair J G, Groth Paul, Goble Carole, Grethe Jeffrey S, Heringa Jaap, 't Hoen Peter A C, Hooft Rob, Kuhn Tobias, Kok Ruben, Kok Joost, Lusher Scott J, Martone Maryann E, Mons Albert, Packer Abel L, Persson Bengt, Rocca-Serra Philippe, Roos Marco, van Schaik Rene, Sansone Susanna-Assunta, Schultes Erik, Sengstag Thierry, Slater Ted, Strawn George, Swertz Morris A, Thompson Mark, van der Lei Johan, van Mulligen Erik, Velterop Jan, Waagmeester Andra, Wittenburg Peter, Wolstencroft Katherine, Zhao Jun, Mons Barend (2016). The FAIR Guiding Principles for scientific data management and stewardship.. Scientific Data.

